# Mesenchymal stem cells therapy improves ovarian function in premature ovarian failure: a systematic review and meta-analysis based on preclinical studies

**DOI:** 10.3389/fendo.2023.1165574

**Published:** 2023-07-06

**Authors:** Congcong Guo, Yubo Ma, Yanqiu Situ, Li Liu, Guoqun Luo, Huan Li, Wenmin Ma, Li Sun, Wen Wang, Qiuying Weng, Linlin Wu, Dazhi Fan

**Affiliations:** ^1^ Reproductive Medicine Center, Foshan Women and Children Hospital, Foshan, Guangdong, China; ^2^ Department of Epidemiology and Biostatistics, School of Public Health, Anhui Medical University, Hefei, Anhui, China; ^3^ Department of Library, The First Affiliated Hospital, College of Medicine, Zhejiang University, Hangzhou, Zhejiang, China; ^4^ Reproductive Medical Center, Zhaoqing Westriver Hospital, Zhaoqing, Guangdong, China; ^5^ Department of Library, Foshan Women and Children Hospital, Foshan, Guangdong, China; ^6^ Department of Obstetrics, Foshan Women and Children Hospital, Foshan, Guangdong, China; ^7^ Foshan Institute of Fetal Medicine, Foshan Women and Children Hospital, Foshan, Guangdong, China

**Keywords:** mesenchymal stem cells, premature ovarian failure, ovarian function, fertility, meta-analysis

## Abstract

**Background:**

Studies have revealed that the transplantation of mesenchymal stem cells (MSCs) might be a potential star candidate for premature ovarian failure (POF) in animal experiments. However, individual studies with a small sample size cannot be used to draw a clear conclusion. Therefore, we conducted a systematic review and meta-analysis to explore the potential of using MSCs in the treatment of POF in animals.

**Methods:**

Seven databases were searched for studies exploring the effect of the transplantation of MSCs on POF in animal models. The PRISMA guideline was followed, and the methodological quality was ensured using SYRCLE’s risk of bias tool. RevMan 5.4 and STATA 12.0 software was performed to meta-analysis.

**Results:**

In total, 37 studies involving 1,079 animals were included. Significant associations were found for MSCs with the levels of E2 (SMD 2.69 [95% CI 1.97, 3.41]), FSH (-2.02, [-2.74, -1.30]), primary follicles (2.04, [1.17, 2.92]), secondary follicles (1.93, [1.05, 2.81]), and primordial follicles (2.38, [1.19, 3.57]. Other outcomes, such as AMH, LH, INHB, antral follicles, growing follicles, mature follicles, and early antral were also found to be significant. There was no difference in FSH/LH, corpus leteum, follicles, and estruc cycle.

**Conclusions:**

Our meta-analysis result indicated that the transplantation of MSCs might exert therapeutic effects on animal models of POF, and these effects might be associated with improving the disorder of the sexual cycle, modulating serum hormone expressions to a better state, and restoring ovarian function.

## Introduction

Premature ovarian failure (POF) is one of the most common causes of female infertility (1). The main feature is ovarian degeneration before the age of 40, with a possibility of ovarian function fluctuating before complete cessation (2). It usually manifests hypoestrogenism, hypergonadotropism, and amenorrhea (1). POF can arise from a range of conditions, including genetic disorders, iatrogenic factors, chemotherapy injury, autoimmunity, and environmental and infectious causes (3). It has an important social and psychological impact, in addition to physiological consequences such as autoimmune disorders, heart disease, and osteoporosis (4). To date, hormone replacement therapy (HRT) is strongly recommended for women with POF, and it can improve hot flushes and vaginal and urinary symptoms for women with POF (5). In addition to HRT, new methods, such as ovarian transplantation, gene therapy, and stem cell therapy, are also urgently needed to improve the treatment efficacy of POF (6).

Mesenchymal stem cells (MSCs) are characterized by multidirectional differentiation potential. They can be isolated from various tissues, such as the peripheral blood, placenta, umbilical cord (UC), adipose tissue (AD), bone marrow (BM), and menstrual fluid (7). Studies reveal that MSC transplantation is a potential star candidate for POF. Due to their safety and manufacturing, the transplantation of MSCs has mainly focused on animal experiments for POF. Researchers found ovarian function could improve after the transplantation of human umbilical cord MSCs (hUC-MSCs) in a POF rat model (8, 9). The transplantation of BM-MSCs can also regain fertility in radiation-associated POF animal models (10). Deng T et al. (11) found that ovarian injury was reduced and ovarian function was improved after UC-MSC transplantation in a chemotherapy-induced POF mice model. In addition, studies have also revealed that Human menstrual blood MSC (HuMenSC) and chorionic plate MSC (CP-MSC) transplantation can be considered suitable treatments for POF (12, 13). It is worth noting that these studies were based on small sample sizes and were highly volatile. Because of the small sample size of each study, the treatment may overstate the efficacy of the individual animal experiments.

Studies need to be systematically combined to reach more robust conclusions. Meta-analysis can obtain stable results by increasing the sample size. There is a phenomenon of inconsistency in recent preclinical studies regarding the treatment of POF using MSC transplantation. Therefore, we conducted a systematic review and meta-analysis of preclinical animal data to evaluate the available evidence for MSCs in the treatment of POF.

## Methods

We followed the PRISMA guideline for this study (**Supplementary Table 1**). **Supplementary Data Sheet 1** shows the protocol. The study was exempt from research ethics board approval.

### Search strategy

We searched three English (PubMed, Embase, and Web of Science) and four Chinese (VIP, Wanfang, CBM, and CNKI) databases from inception to August 2022. Searches included terms relating to mesenchymal stem cells, premature ovarian failure, and animal models (**Supplementary Data Sheet 2**). Study language was not a restriction. With the help of two medical librarians (LL and LS), two authors (CG and TS) independently searched and screened the databases.

### Study selection

We considered studies if they explored the effect of MSC transplantation on POF in animal models. The animal consisted of rats, mice, rabbits, and other animals. The exposure was MSC transplantation, including both human and animal MSCs, from umbilical cord MSCs (UC-MSCs), bone marrow MSCs (BMMSCs), menstrual blood MSCs (MenSCs), placenta MSCs (PMSCs), chorionic plate MSCs (CPMSCs), adipose MSCs (ADMSCs), amniotic fluid MSCs (AFMSCs), and other tissues-derived MSCs. The control was placebo transplantation, such as 0.9% saline and phosphate-buffered saline.

The primary outcomes were estradiol (E2), follicle-stimulating hormone (FSH), primary follicles, and secondary follicles. The secondary outcomes were other sex hormone outcomes, such as luteinizing hormone (LH), anti-Mullerian hormone (AMH), FSH/LH, inhibin B (INHB), and other follicle outcomes, such as primordial follicles, growing follicles, antral follicles, mature follicles, atretic follicles, corpus leteum, follicles, early antral, and estruc cycle. The type of study included was randomized controlled studies. We excluded case studies, observational studies, reviews, and human studies. If they were duplicates, studies with the most complete information were included in the analysis.

### Data extraction

Using pretested forms, two authors (CG and TS) independently screened articles for eligibility, and extracted data. Study author, publication year, author country, sample size, type and dose of MSCs, route of transplantation, type of animals, and assessment of outcomes were extracted. The closest to 4-week outcomes were extracted when results for more than one time point appeared. Conflicts were resolved through consensus with a third author (DF).

### Quality assessment

The methodological quality of each included study was assessed with the use of SYRCLE’s risk of bias tool (14). The contents of the evaluation covered 10 entries in six aspects (**Supplementary Table 2**). Each entry was categorized as “Yes”, “No”, or “Unclear” regarding bias. Two reviewers (CG and TS) independently assessed the quality of each included study and were mediated by the third reviewer (DF).

### Data analysis

Data analysis and forest plots were combined for meta-analysis using Review Manager 5.4.1 and Stata version 12. Standardized mean difference (SMD) with a 95% confidence interval (95% CI) was approved for each of the included studies. *I^2^* statistics (> 50% indicating substantial heterogeneity) assessed the variation study-to-study. If substantial heterogeneity existed, a random effects model was used. Otherwise, a fixed effects model was applied. A Funnel plot and Begg’s and Egger’s tests were provided to assess the potential publication biases if more than 10 studies for one outcome were included. We performed subgroup analyses for animal species (rats, mice, rabbits, or mice and rats), induced (chemotherapy, autoimmune, or radiation), source of MSCs (human or animal), types of MSCs (UCMSCs, BMMSCs, AFMSCs, CPMSCs, MenSCs, ADMSCs, or PMSCs), the transplantation dose (10^5^, 10^6^, or 10^7^), and route of transplantation (intravenously injected or intra ovary injected). We considered *p*-values of less than 0.05 to be statistically significant.

## Results

### Search results and characteristics

**Figure 1:**
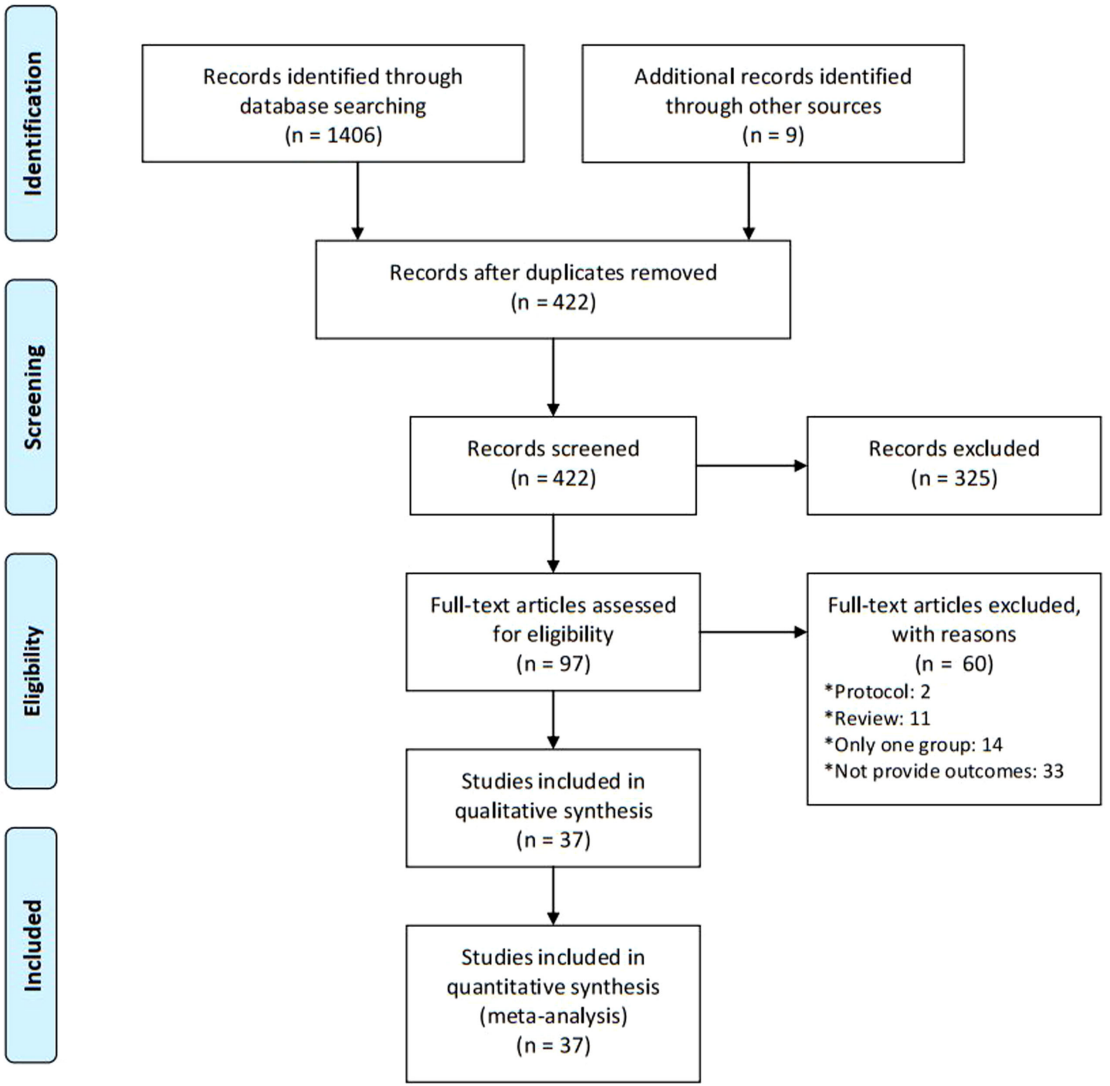
Flow diagram of the study selection process for this systematic review and meta-analysis.

Of 1,406 citations identified, we assessed 97 full articles. Eventually, 37 studies, which included 1,079 animals (540 in the MSC group and 539 in the control group), proved eligible (**Figure 1**). The animal species included rats (n = 23) (8–10, 12, 15–33), mice (n = 12) (11, 13, 34–43), and rabbits (n = 2) (44, 45). Most MSCs were from the umbilical cord (UCMSCs, n = 18) (8, 9, 11, 16, 18, 19, 21, 25, 27, 29, 34, 36–39, 42, 44, 45) or bone marrow (BMMSCs, n = 11) (10, 17, 22–24, 28, 30, 31, 33, 41, 43), and, other MSCs were from menstrual blood (MenMSCs, n = 2) (12, 35), placenta (PMSCs, n = 2) (20, 40), adipose (ADMSCs, n = 2) (15, 26), amniotic fluid (AFMSCs, n = 1) (32), or chorionic plate (CPMSCs, n = 1) (13). In total, 24 MSCs were from human tissues, and the other 13 MSCs were from animal tissues. Thirty-one POF models were induced by chemotherapy, five models were induced by autoimmune, and one model was induced by radiotherapy. Six studies transplanted 10^5^ cells, 27 studies transplanted 10^6^ cells, and four studies transplanted 10^7^ cells. Furthermore, 25 studies used intravenous (IV) injections to transplant cells, and 12 studies used intra-ovary injections (IO). Outcomes were sex hormones, including E2 (n = 30), FSH (n = 27), AMH (n = 7), LH (n = 6), FSH/LH (n = 5), and INHB (n = 2), ovarian follicles counting, including primary follicles (n = 13), secondary follicles (n = 12), primordial follicles (n = 11), atretic follicles (n = 9), antral follicles (n = 6), growing follicles (n = 5), mature follicles (n = 5), corpus leteum (n = 5), follicles (n =3), and early antral (n = 2), and estrus cycle (n = 7) (**Table 1**).

**Table 1:** Characteristics of the included studies.

Author, year	Cases (SC/control)	Mode of POF induction	Type of Animal	Type of MSCs (source)	Cell dose/control	Route of transplantation	Follow-up	Outcomes
Yan PY, 2022 (15)	10/10	Chemotherapy-Induced	SD rat	ADMSCs (rat)	5×10^5^/0.9% saline	IV	28 days	LH, FSH, E_2_
Zhao SY, 2022 (34)	14/14	Chemotherapy-Induced	ICR mice	UCMSCs (human)	2×10^6^/0.9% saline	IV	14 days	FSH, E_2_, AMH, PrF, PF, SF, AF
Wu Y, 2021 (16)	9/6	Autoimmune-Induced	SD rat	UCMSCs (human)	2×10^6^/0.9% saline	IO	21 days	PrF, PF, SF, MF, CL
Deng TR, 2021 (11)	8/8	Chemotherapy-Induced	C57BL/6 mice	UCMSCs (human)	1×10^6^/PBS	IV	28 days	FSH, E_2_, EC, PF, SF, AF
El-Derany MO, 2021 (10)	30/30	Radiation-Induced	SD rat	BMMSCs (rat)	2×10^6^/PBS	IV	21 days	FSH, E_2_, EC
Zhang L, 2021 (44)	5/5	Chemotherapy-Induced	rabbit	UCMSCs (human)	3×10^7^/0.9% saline	IV	28 days	FSH, FSH/LH, E_2_, INHB, AMH, GF, AF, CL
Zhang XY, 2021 (8)	20/20	Autoimmune-Induced	SD rat	UCMSCs (human)	1×10^6^/PBS	IO	20 days	LH, FSH, E_2_, EC, NF
Li Q, 2020 (17)	12/12	Chemotherapy-Induced	SD rat	BMMSCs (rat)	1×10^6^/0.9% saline	IV	45 days	FSH, E_2_, PrF, GF, MF, AF
Wang Z, 2020 (9)	15/15	Autoimmune-Induced	SD rat	UCMSCs (human)	4×10^6^/PBS	IV	21 days	PrF, PF, SF, MF, CL
Xu YY, 2020 (35)	16/16	Chemotherapy-Induced	C57BL/6 mice	MenSCs (human)	4×10^6^/PBS	IV	14 days	E_2_
Zhang J, 2020 (45)	5/5	Chemotherapy-Induced	rabbit	UCMSCs (human)	3×10^7^/0.9% saline	IV	28 days	FSH, E_2_
Hou QN, 2019 (20)	15/15	Chemotherapy-Induced	SD rat	PMSCs (human)	2×10^7^/0.9% saline	IV	15 days	PF, SF, MF, AF
Lin HW, 2019 (36)	12/12	Chemotherapy-Induced	BALB/c mice	UCMSCs (human)	4×10^5^/0.9% saline	IV	7 days	LH, FSH, E_2_,
Manshadi MD, 2019 (12)	6/6	Chemotherapy-Induced	Wistar rat	MenSCs (human)	1×10^6^/PBS	IV	28 days	E2
Tan L, 2019 (19)	10/10	Chemotherapy-Induced	SD rat	UCMSCs (human)	1×10^6^/0.9% saline	IV	17 days	LH, FSH, E_2_, GF, AF, CL
Yang YJ, 2019 (37)	15/15	Chemotherapy-Induced	C57BL/6 mice	UCMSCs (human)	2×10^5^/PBS	IO	28 days	FSH, E_2_, AMH, PrF, PF, SF, AnF, EC
Zheng Q, 2019 (18)	12/12	Chemotherapy-Induced	SD rat	UCMSCs (human)	5×10^6^/PBS	IV	14 days	EC, PrF, PF, SF, EA
Zhao Y, 2019 (38)	15/15	Chemotherapy-Induced	CD-1 mice	UCMSCs (human)	2×10^6^/0.9% saline	IV	21 days	FSH, E_2_, AMH, PrF, GF, AF
Zhuang YQ, 2019 (39)	12/12	Chemotherapy-Induced	ICR mice	UCMSCs (human)	1×10^6^/sterilizing water	IV	14 days	FSH/LH, AMH, PF, SF, PrF, AnF
Li J, 2018 (13)	25/25	Chemotherapy-Induced	C57BL/6 mice	CPMSCs (human)	8×10^6^/0.9% saline	IV	28 days	FSH, E_2_, EC
Li XR, 2018 (23)	20/20	Chemotherapy-Induced	Wistar rat	BMMSCs (rat)	2×10^6^/PBS	IO	30 days	FSH, E_2_
Wang LL, 2018 (22)	25/25	Chemotherapy-Induced	Wistar rat	BMMSCs (rat)	1×10^6^/PBS	IO	30 days	FSH, E_2_, NF, PF, SF, AnF, PrF
Yin N, 2018 (40)	17/18	Autoimmune-Induced	BALB/c mice	PMSCs (human)	1×10^6^/PBS	IV	14 days	FSH, E_2_, PF, SF
Zhang LL, 2018 (21)	22/22	Chemotherapy-Induced	SD rat	UCMSCs (human)	1×10^6^/sterilizing water	IV	17 days	FSH/LH, AMH, INHB, GF, AF, CL
Badawy A, 2017 (41)	10/10	Chemotherapy-Induced	BALB/c mice	BMMSCs (mice)	5×10^5^/0.9% saline	IV	21 days	FSH, E_2_
Jia XC, 2017 (25)	24/24	Chemotherapy-Induced	SD rat	UCMSCs (human)	6×10^6^/0.9% saline	IV	32 days	E_2_, PF, MF
Wu Q, 2017 (24)	12/12	Chemotherapy-Induced	SD rat	BMMSCs (rat)	3×10^6^/0.9% saline	IV	28 days	FSH, E_2_, LH
Elfayomy AK, 2016 (29)	12/15	Chemotherapy-Induced	Wistar rat	UCMSCs (human)	2×10^6^/0.9% saline	IO	28 days	FSH, E_2_, AnF
Gabr H, 2016 (28)	12/12	Chemotherapy-Induced	Albino rat	BMMSCs (rat)	1×10^6^/0.9% saline	IV	14 days	FSH, E_2_,
Song D, 2016 (27)	7/7	Chemotherapy-Induced	Wistar rat	UCMSCs (human)	1×10^5^/0.9% saline	IO	28 days	FSH, E_2_, AMH, EF, PF, SF, PrF
Su J, 2016 (26)	16/14	Chemotherapy-Induced	SD rat	ADMSCs (rat)	2×10^6^/PBS	IO	28 days	E_2_, AnF
Qin JJ, 2015 (31)	12/12	Chemotherapy-Induced	SD rat	BMMSCs (rat)	3×10^6^/0.9% saline	IV	28 days	FSH, E_2_,
Ye XF, 2015 (30)	22/22	Chemotherapy-Induced	Wistar rat	BMMSCs (rat)	1×10^7^/0.9% saline	IO	30 days	FSH, E_2_, NF,
Fu XF, 2013 (42)	20/20	Autoimmune-Induced	BALB/c mice	UCMSCs (human)	2×10^6^/0.9% saline	IO	30 days	FSH, E_2_, SF, PF, AnF, PrF
Li J, 2012 (32)	8/8	Chemotherapy-Induced	SD rat	AFMSCs (human)	2×10^6^/0.9% saline	IV	7 days	FSH, E_2_
Wang Y, 2011 (43)	15/15	Chemotherapy-Induced	C57 mice	BMMSCs (mice)	2×10^5^/PBS	IO	28 days	FSH, LH,
Fu X, 2008 (33)	20/20	Chemotherapy-Induced	Wistar rat	BMMSCs (rat)	2×10^6^/0.9% saline	IO	28 days	FSH, E_2_

UC-MSCs, umbilical cord mesenchymal stem cells; BMMSCs, bone marrow mesenchymal stem cells; MenSCs, Menstrual blood mesenchymal stem cells; PMSCs, placenta mesenchymal stem cells; CPMSCs, chorionic plate mesenchymal stem cells; ADMSCs, adipose mesenchymal stem cell; AFMSCs, amniotic fluid mesenchymal stem cells; IV, intravenously injected; IO, intra ovary injected; POF, premature ovarian failure; SD rat, Sprague-Dawley rat; pZP3, zona pellucida glycoprotein 3; PBS, phosphate-buffered saline; LH, luteinizing hormone; FSH, follicle-stimulating hormone; E_2_, estradiol; AMH, anti Mullerian hormone; INHB, inhibin B; EC, estrus cycle; NF, number of follicles; GF, growing follicle; AF, atretic follicles; CL, corpora luteum; AnF, antral follicles; PrF, primordial follicles; PF, primary follicles; SF, secondary follicles; MF, mature follicles; EF, early antral.

### Assessment of study quality and risk of bias

The quality and risk of bias for studies included in the meta-analysis are presented in **Supplementary Table 2**. Only six of the 37 studies mentioned sequence generation. The baseline characteristics were comparability. Allocation concealment, attrition, reporting, and other bias were not found. Performance bias and detection bias were unclear for all the included studies.

### Meta-analysis results

#### E2

**Figure 2:**
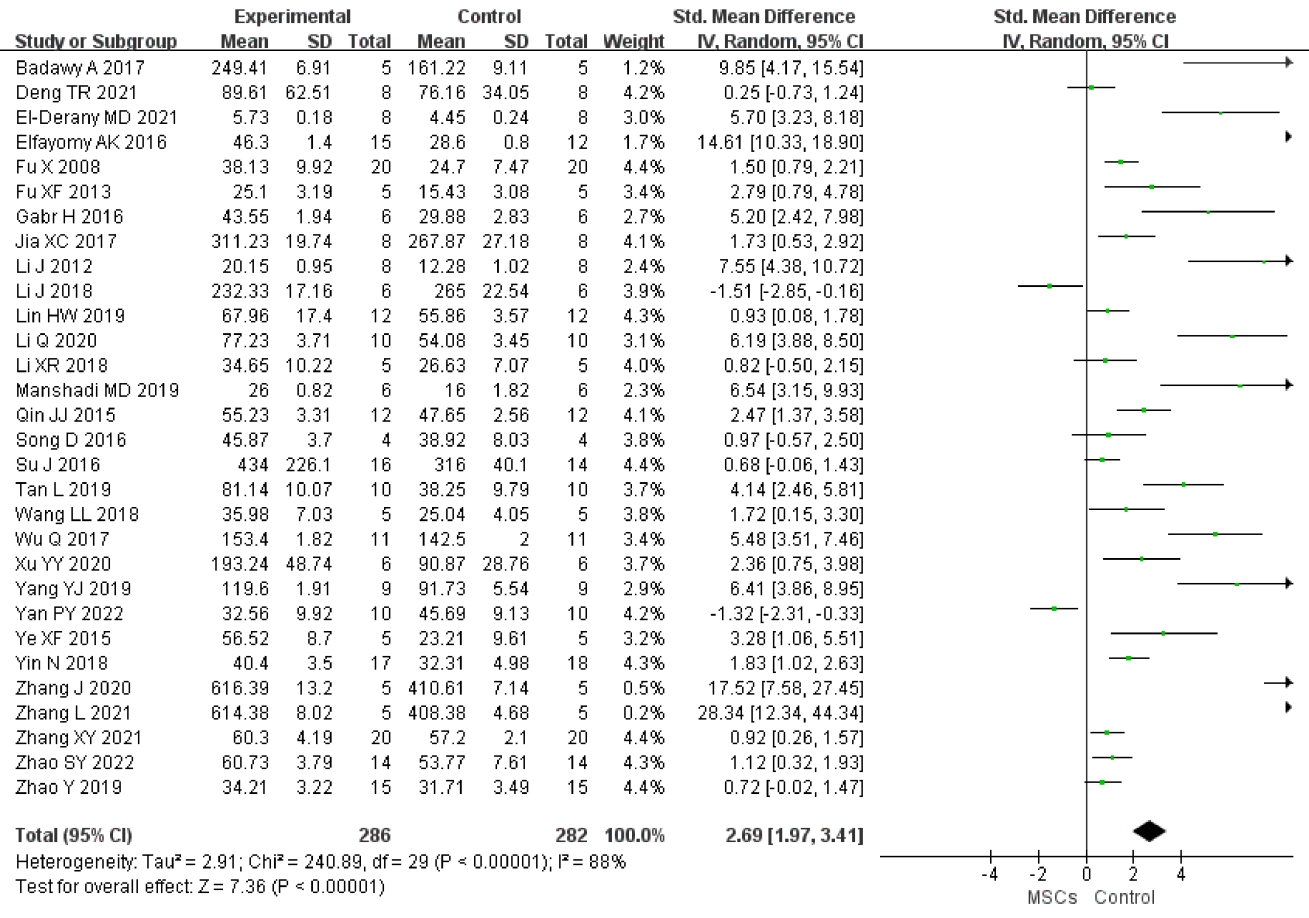
Forest plot summarizing the effects of MSCs on E2 in preclinical models of POF.

In total, 30 studies (n = 568 animals) provided data for this comparison (286 vs. 282 animals). E2 was noted to be higher in the MSC group (SMD 2.69, 95% CI 1.97 to 3.41) in the random-effect model (*I^2 ^= *88) (**Figure 2**). A funnel plot showed asymmetry (**Supplementary Figure 1**), and Begg’s (z = 4.46, p = 0.001) and Egger’s (t = 6.50, p = 0.001) tests also indicated that there was a statistical difference.

#### FSH

**Figure 3:**
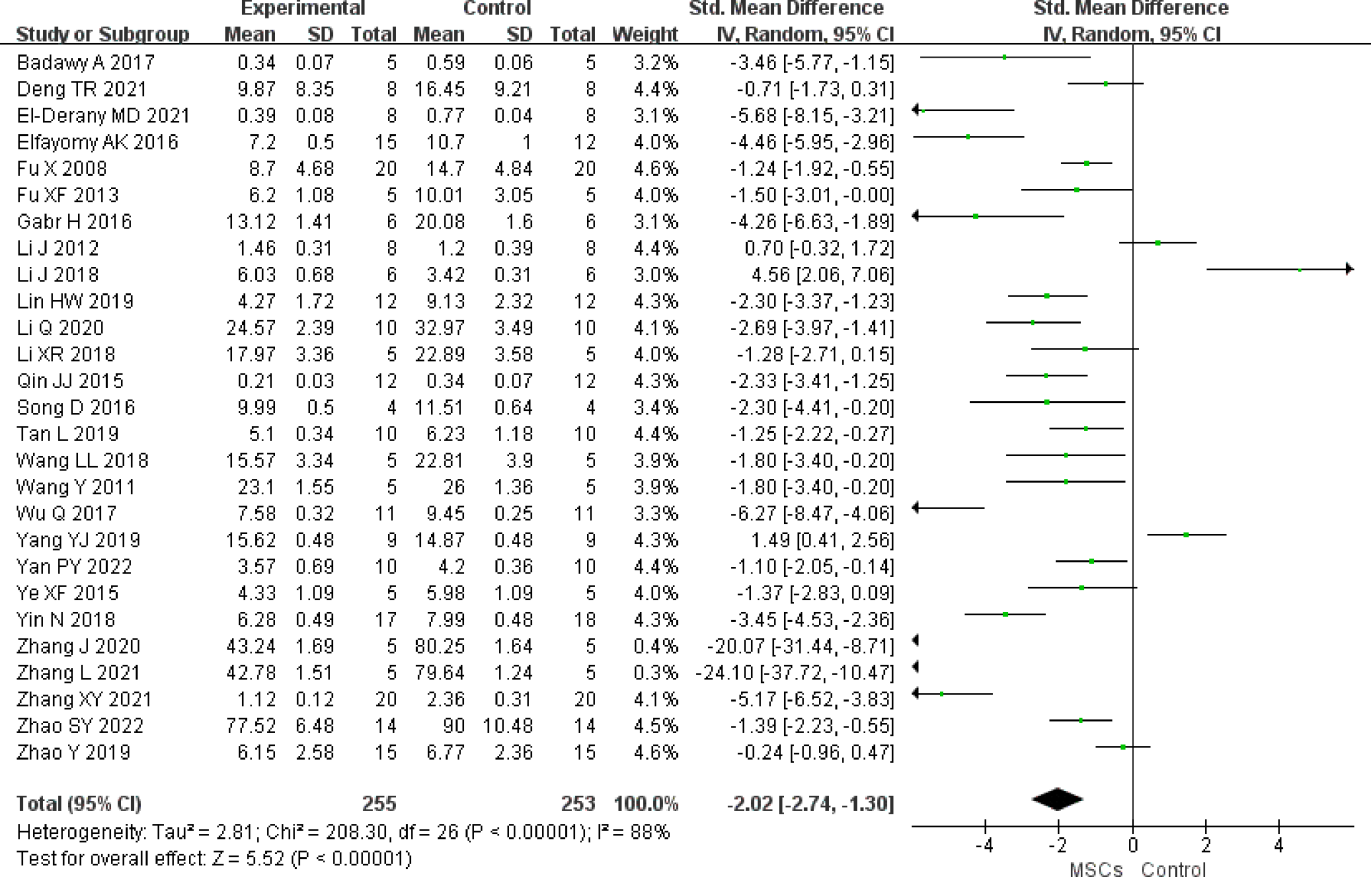
Forest plot summarizing the effects of MSCs on FSH in preclinical models of POF.

In total, 27 studies (n = 508 animals) provided data for FSH comparison (255 vs. 253 animals). FSH was noted to be lower in the MSC group (SMD -2.02, 95% CI -2.74to -1.30) in the random-effect mode (*I^2 ^= *88%) (**Figure 3**). A funnel plot showed asymmetry (**Supplementary Figure 2**), and Begg’s (z = 3.21, p = 0.001) and Egger’s (t = -2.96, p = 0.007) tests also indicated that there was a statistical difference.

#### Primary follicles

**Figure 4:**
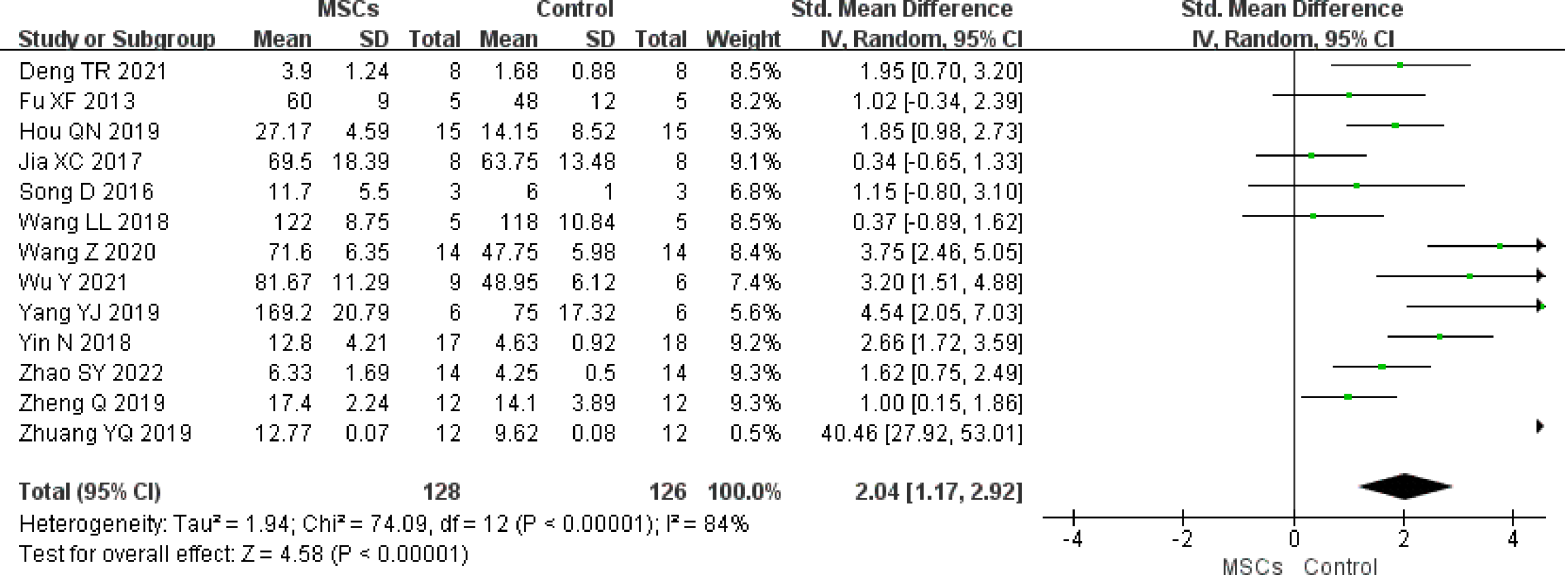
Forest plot summarizing the effects of MSCs on primary follicles in preclinical models of POF.

In total, 13 studies (n = 254 animals) reported primary follicles. It was noted to be higher in the MSC group (SMD 2.04, 95% CI 1.17 to 2.92) in the random-effect mode (*I^2 ^= *84%) (**Figure 4**). A funnel plot showed asymmetry (**Supplementary Figure 3**), and Begg’s (z = 1.77, p = 0.077) and Egger’s (t = 3.10, p = 0.010) tests also indicated that there was a statistical difference.

#### Secondary follicles

**Figure 5:**
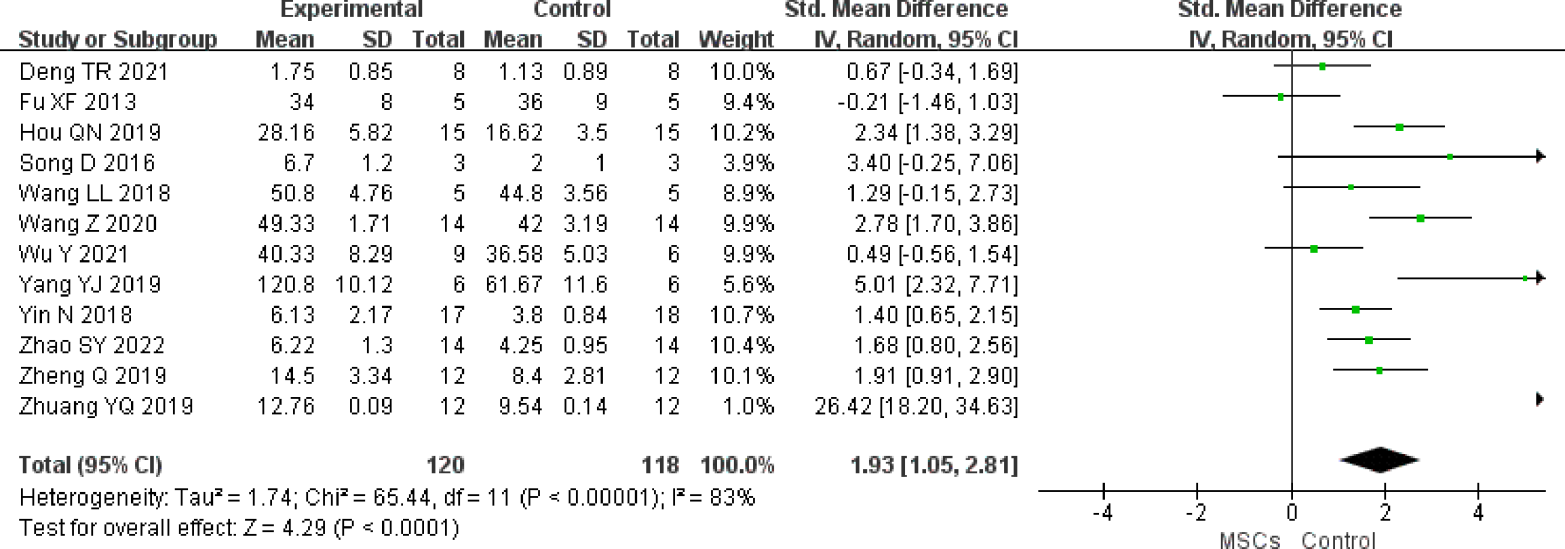
Forest plot summarizing the effects of MSCs on secondary follicles in preclinical models of POF.

The effect size for secondary follicles was pooled from 12 studies (n = 238 animals). A significant association was found between MSC therapy and an increase in the secondary follicle counts (SMD 1.93, (95% CI 1.05 to 2.81) in the random-effect mode (*I^2 ^= *83%) (**Figure 5**). A funnel plot showed symmetry (**Supplementary Figure 4**), and Begg’s (z = 1.17, p = 0.244) and Egger’s (t = 2.72, p = 0.022) tests also indicated that there was a statistical difference.

#### Primordial follicles

**Figure 6:**
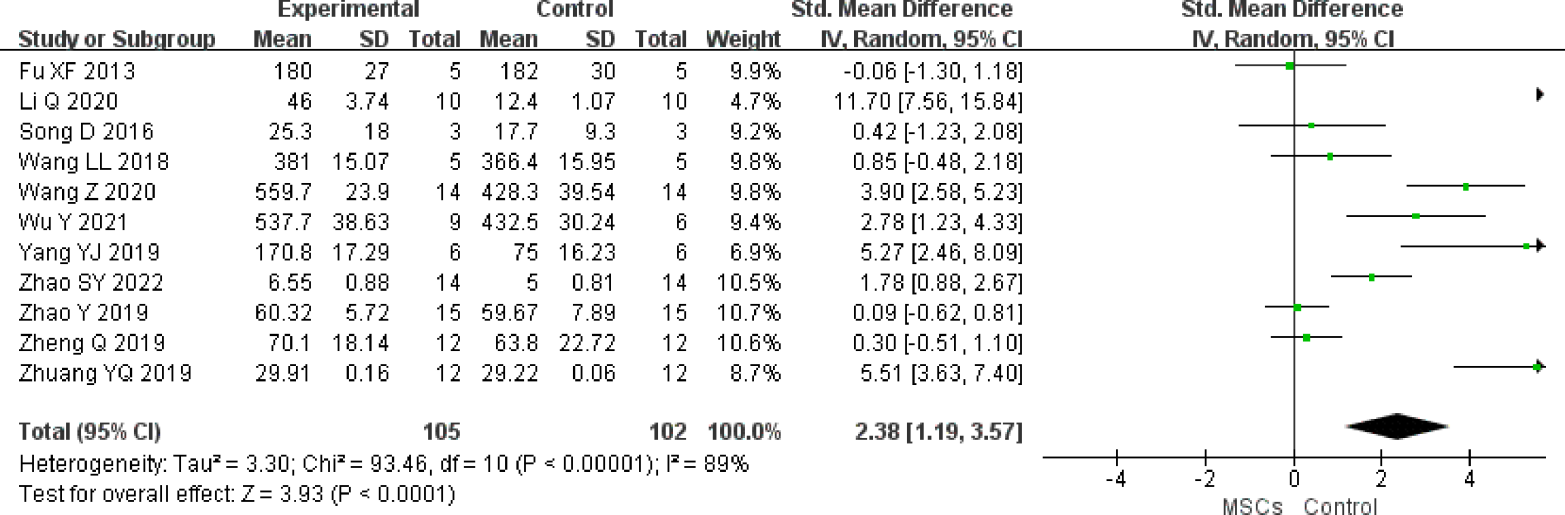
Forest plot summarizing the effects of MSCs on primordial follicles in preclinical models of POF.

We identified 11 studies (n = 207 animals) that compared MSC treatment with controls for primordial follicles. Higher primordial follicle counts were noted in the MSC group (SMD 2.38, 95% CI 1.19 to 3.57) in the random-effect mode (*I^2 ^= *89%) (**Figure 6**). A funnel plot showed asymmetry (**Supplementary Figure 5**), and Begg’s (z = 2.65, p = 0.008) and Egger’s (t = 3.75, p = 0.005) tests also indicated that there was a statistical difference.

### Other outcomes

The meta-analysis showed that MSCs significantly increased AMH (seven studies, n = 130 animals, SMD 1.41 95% CI 0.67 to 2.14, *I^2 ^= *67%, random-effect mode), INHB (two studies, n = 20 animals, SMD 23.51 95% CI 14.11 to 32.91, *I^2 ^= *0, fixed-effect mode), antral follicles (six studies, n = 113 animals, SMD 2.85 95% CI 0.58 to 5.11, *I^2 ^= *93%, random-effect mode), growing follicles (five studies, n = 116 animals, SMD 2.08 95% CI 1.19 to 2.98, *I^2 ^= *68%, random-effect mode), mature follicles (five studies, n = 109 animals, SMD 3.10 95% CI 1.86 to 4.34, *I^2 ^= *76%, random-effect mode), and early antral (two studies, n = 30 animals, SMD 1.00 95% CI 0.23 to 1.78, *I^2 ^= *0, fixed-effect mode), and significantly reduced LH (six studies, n = 132 animals, SMD -2.53 95% CI -3.79 to -1.27, *I^2 ^= *85%, random-effect mode), atretic follicles (nine studies, n = 184 animals, SMD -1.71 95% CI -3.08 to -0.33, *I^2 ^= *90%, random-effect mode). However, there was no difference in FSH/LH (three studies, n = 46 animals, SMD -0.08 95% CI -0.66 to 0.49, *I^2 ^= *0, fixed-effect mode), corpus leteum (five studies, n = 85 animals, SMD 1.02 95% CI -1.47 to 3.51, *I^2 ^= *93%, random-effect mode), follicles (three studies, n = 60 animals, SMD 3.05 95% CI -0.58 to 6.69, *I^2 ^= *94%, random-effect mode), and estruc cycle (seven studies, n = 180 animals, SMD 0 95% CI -1.65 to 1.66, *I^2 ^= *94%, random-effect mode) between the MSC group and control group.

### Subgroup analysis

Most of the subgroup analyses did not change substantially, except for a few variables that resulted from fewer data (**Table 2**).

**Table 2:** Results of analysis and subgroup analysis.

Outcomes		No. studies	No. Participants		Summary SMD (95%CI)		*I^2^* (%)
			MSCs	controls	Random	Fixed	
**E2**		30	286	282	2.69 (1.97 to 3.41)	1.38 (1.16 to 1.60)	88
	Animal Species						
	Rats	18	179	174	3.16 (2.16 to 4.16)	1.57 (1.28 to 1.86)	90
	Mice	10	97	98	1.55 (0.64 to 2.46)	1.09 (0.75 to 1.43)	83
	Mice and Rats	28	276	272	2.53 (1.84 to 3.23)	1.37 (1.14 to 1.59)	88
	Rabbits	2	10	10	21.03 (11.10 to 30.97)	20.53 (12.09 to 28.97)	21
	Induced						
	Chemotherapy	26	236	231	2.81 (1.97 to 3.65)	1.34 (1.09 to 1.59)	88
	Autoimmune	3	42	43	1.56 (0.67 to 2.46)	1.37 (0.88 to 1.86)	60
	Radiation	1	8	8	5.70 (3.23 to 8.18)	5.70 (3.23 to 8.18)	
	Source of MSCs						
	Human	18	173	171	2.61 (1.69 to 3.54)	1.32 (1.04 to 1.60)	88
	Animal	12	113	111	2.85 (1.63 to 4.08)	1.47 (1.11 to 1.84)	89
	Types of MSCs						
	UCMSCs	13	130	127	2.55 (1.50 to 3.60)	1.25 (0.94 to 1.57)	88
	BMMSCs	10	87	87	3.59 (2.33 to 4.84)	2.38 (1.92 to 2.84)	82
	AFMSCs	1	8	8	7.55 (4.38 to 10.72)	7.55 (4.38 to 10.72)	
	CPMSCs	1	6	6	-1.51 (-2.85 to -0.16)	-1.51 (-2.85 to -0.16)	
	MenSCs	2	12	12	4.17 (0.12 to 8.23)	3.14 (1.68 to 4.59)	79
	ADMSCs	2	26	24	-0.29 (-2.25 to 1.67)	-0.04 (-0.63 to 0.56)	90
	PMSCs	1	17	18	1.83 (1.02 to 2.63)	1.83 (1.02 to 2.63)	
	Dose of transplantation						
	10^5^	3	15	15	14.82 (0.21 to 29.42)	4.40 (2.25 to 6.55)	88
	10^6^	22	231	227	2.59 (1.85 to 3.32)	1.49 (1.25 to 1.73)	87
	10^7^	5	40	40	2.34 (0.03 to 4.64)	0.54 (-0.03 to 1.12)	91
	Route of transplantation						
	IV	20	182	183	2.91 (1.91 to 3.91)	1.40 (1.11 to 1.68)	89
	IO	10	104	99	2.40 (1.35 to 3.46)	1.35 (1.00 to 1.70)	86
**FSH**		27	255	253	-2.02 (-2.74 to -1.30)	-1.49 (-1.73 to -1.26)	88
	Animals						
	Rats	15	149	146	-2.52 (-3.41 to -1.63)	-1.87 (-2.19 to -1.56)	86
	Mice	10	96	97	-0.97 (-2.05 to 0.11)	-1.00 (-1.35 to -0.65)	88
	Mice and Rats	25	245	243	-1.89 (-2.58 to -1.20)	-1.48 (-1.72 to -1.24)	87
	Rabbits	2	10	10	-21.73 (-30.46 to -13.00)	-21.73 (-30.46 to -13.00)	0
	Induced						
	Chemotherapy	23	205	202	-1.66 (-2.37 to -0.94)	-1.22 (-1.47 to -0.97)	85
	Autoimmune	3	42	43	-3.40 (-5.30 to -1.50)	-3.50 (-4.23 to -2.76)	84
	Radiation	1	8	8	-5.68 (-8.15 to -3.21)	-5.68 (-8.15 to -3.21)	
	Source of MSCs						
	Human	15	153	151	-1.61 (-2.74 to -0.47)	-1.21 (-1.51 to -0.90)	91
	Animal	12	102	102	-2.45 (-3.22 to -1.68)	-1.92 (-2.29 to -1.55)	72
	Types of MSCs						
	UCMSCs	12	122	119	-2.07 (-3.27 to -0.88)	-1.31 (-1.64 to -0.97)	90
	BMMSCs	11	92	92	-2.63 (-3.48 to -1.78)	-2.07 (-2.47 to -1.66)	72
	AFMSCs	1	8	8	0.70 (-0.32 to 1.72)	0.70 (-0.32 to 1.72)	
	CPMSCs	1	6	6	4.56 (2.06 to 7.06)	4.56 (2.06 to 7.06)	
	PMSCs	1	17	18	-3.45 (-4.53 to -2.36)	-3.45 (-4.53 to -2.36)	
	ADMSCs	1	10	10	-1.10 (-2.05 to -0.14)	-1.10 (-2.05 to -0.14)	
	Dose of transplantation						
	10^5^	6	45	45	-1.45 (-2.88 to -0.01)	-1.04 (-1.57 to -0.52)	85
	10^6^	18	195	193	-2.07 (-2.91 to -1.23)	-1.60 (-1.87 to -1.33)	89
	10^7^	3	15	15	-14.22 (-30.74 to 2.30)	-1.92 (-3.36 to -0.48)	90
	Route of transplantation						
	IV	17	162	163	-2.11 (-3.05 to -1.17)	-1.45 (-1.75 to -1.16)	88
	IO	10	93	90	-1.91 (-3.11 to -0.71)	-1.57 (-1.96 to -1.18)	88
**Primary follicles**		13	128	126	2.04 (1.17 to 2.92)	1.71 (1.39 to 2.04)	86
	Animals						
	Rats	7	66	63	1.61 (0.68 to 2.54)	1.45 (1.03 to 1.88)	77
	Mice	6	62	63	2.96 (1.18 to 4.74)	2.09 (1.58 to 2.60)	89
	Mice and Rats	13	128	126	2.04 (1.17 to 2.92)	1.71 (1.39 to 2.04)	86
	Induced						
	Chemotherapy	9	83	83	1.76 (0.66 to 2.87)	1.35 (0.96 to 1.73)	85
	Autoimmune	4	45	43	2.65 (1.55 to 3.74)	2.65 (2.03 to 3.26)	65
	Source of MSCs						
	Human	12	123	121	2.20 (1.28 to 3.12)	1.81 (1.47 to 2.15)	84
	Animal	1	5	5	0.37 (-0.89 to 1.62)	0.37 (-0.89 to 1.62)	
	Types of MSCs						
	UCMSCs	10	91	88	2.29 (1.11 to 3.47)	1.65 (1.25 to 2.05)	86
	BMMSCs	1	5	5	0.37 (-0.89 to 1.62)	0.37 (-0.89 to 1.62)	
	AFMSCs	2	32	33	2.24 (1.45 to 3.02)	2.23 (1.59 to 2.87)	34
	Dose of transplantation						
	10^5^	2	9	9	2.76 (-0.56 to 6.08)	2.44 (0.91 to 3.98)	77
	10^6^	10	104	102	2.01 (0.94 to 3.08)	1.65 (1.29 to 2.01)	87
	10^7^	1	15	15	1.85 (0.98 to 2.73)	1.85 (0.98 to 2.73)	
	Route of transplantation						
	IV	8	100	101	2.18 (1.01 to 3.34)	1.77 (1.40 to 2.13)	88
	IO	5	28	25	1.86 (0.49 to 3.22)	1.51 (0.79 to 2.23)	70
**Secondary follicles**		12	120	118	1.93 (1.05 to 2.81)	1.56 (1.23 to 1.89)	85
	Animals						
	Rats	6	58	55	1.85 (1.09 to 2.62)	1.85 (1.38 to 2.33)	57
	Mice	6	62	63	2.39 (0.70 to 4.09)	1.29 (0.84 to 1.75)	90
	Mice and Rats	12	120	118	1.93 (1.05 to 2.81)	1.56 (1.23 to 1.89)	85
	Induced						
	Chemotherapy	8	75	75	3.06 (1.38 to 4.75)	1.83 (1.38 to 2.27)	85
	Autoimmune	4	45	43	1.35 (-0.12 to 2.83)	1.24 (0.75 to 1.89)	80
	Source of MSCs						
	Human	11	115	113	2.01 (1.06 to 2.97)	1.58 (1.24 to 1.91)	85
	Animal	1	5	5	1.29 (-0.15 to 2.73)	1.29 (-0.15 to 2.73)	
	Types of MSCs						
	UCMSCs	9	83	80	2.25 (0.97 to 3.54)	1.49 (1.08 to 1.90)	87
	BMMSCs	1	5	5	1.29 (-0.15 to 2.73)	1.29 (-0.15 to 2.73)	
	PMSCs	2	32	33	1.82 (0.90 to 2.73)	1.76 (1.17 to 2.35)	56
	Dose of transplantation						
	10^5^	2	9	9	4.45 (2.28 to 6.62)	4.45 (2.28 to 6.62)	0
	10^6^	9	96	94	1.60 (0.60 to 2.59)	1.38 (1.02 to 1.73)	85
	10^7^	1	15	15	2.34 (1.38 to 3.29)	2.34 (1.38 to 3.29)	
	Route of transplantation						
	IV	7	92	93	2.21 (1.09 to 3.32)	1.79 (1.41 to 2.17)	87
	IO	5	28	25	1.47 (0.03 to 2.91)	0.84 (0.17 to 1.50)	73
**Primordial follicles**		11	105	102	2.38 (1.19 to 3.57)	1.25 (0.89 to 1.61)	89
	Animals						
	Rats	6	53	50	2.65 (0.79 to 4.51)	1.46 (0.93 to 2.00)	90
	Mice	5	52	52	2.22 (0.44 to 4.01)	1.08 (0.59 to 1.56)	91
	Mice and Rats	11	105	102	2.38 (1.19 to 3.57)	1.25 (0.89 to 1.61)	89
	Induced						
	Chemotherapy	8	77	77	2.47 (1.04 to 3.89)	1.04 (0.64 to 1.45)	90
	Autoimmune	3	28	25	2.19 (-0.27 to 4.66)	2.04 (1.25 to 2.82)	90
	Source of MSCs						
	Human	9	90	87	2.01 (0.83 to 3.18)	1.20 (0.82 to 1.57)	88
	Animal	2	15	15	6.09 (-4.54 to 16.72)	1.86 (0.60 to 3.13)	96
	Types of MSCs						
	UCMSCs	9	90	87	2.01 (0.83 to 3.18)	1.20 (0.82 to 1.57)	88
	BMMSCs	2	15	15	6.09 (-4.54 to 16.72)	1.86 (0.60 to 3.13)	96
	Dose of transplantation						
	10^5^	2	9	9	2.71 (-2.03 to 7.45)	1.67 (0.24 to 3.09)	88
	10^6^	9	96	93	2.36 (1.06 to 3.65)	1.22 (0.85 to 1.59)	91
	Route of transplantation						
	IV	6	77	77	3.12 (1.30 to 4.94)	1.29 (0.87 to 1.71)	93
	IO	5	28	25	1.57 (0.08 to 3.05)	1.14 (0.45 to 1.82)	77
**AMH**		7	65	65	1.41 (0.67 to 2.14)	1.29 (0.89 to 1.70)	67
	Animals						
	Rats	2	10	10	0.81 (-0.14 to 1.75)	0.81 (-0.14 to 1.75)	0
	Mice	4	50	50	1.68 (0.54 to 2.81)	1.40 (0.94 to 1.87)	82
	Mice and Rats	6	60	60	1.41 (0.58 to 2.24)	1.28 (0.87 to 1.70)	72
	Rabbits	1	5	5	1.41 (-0.06 to 2.89)	1.41 (-0.06 to 2.89)	
	Induced						
	Chemotherapy	7	65	65	1.41 (0.67 to 2.14)	1.29 (0.89 to 1.70)	67
	Source of MSCs						
	Human	7	65	65	1.41 (0.67 to 2.14)	1.29 (0.89 to 1.70)	67
	Types of MSCs						
	UCMSCs	7	65	65	1.41 (0.67 to 2.14)	1.29 (0.89 to 1.70)	67
	Dose of transplantation						
	10^5^	2	13	13	0.88 (0.06 to 1.71)	0.88 (0.06 to 1.71)	0
	10^6^	4	47	47	1.66 (0.46 to 2.86)	1.42 (0.94 to 1.91)	82
	10^7^	1	5	5	1.41 (-0.06 to 2.89)	1.41 (-0.06 to 2.89)	
	Route of transplantation						
	IV	5	52	52	1.61 (0.62 to 2.60)	1.42 (0.96 to 1.89)	76
	IO	2	13	13	0.88 (0.06 to 1.71)	0.88 (0.06 to 1.71)	0
**LH**		6	68	68	-2.53 (-3.79 to -1.27)	-2.04 (-2.50 to -1.58)	85
	Animals						
	Rats	4	51	51	-3.16 (-4.97 to -1.35)	-2.50 (-3.08 to -1.92)	89
	Mice	2	17	17	-1.24 (-2.01 to -0.48)	-1.24 (-2.01 to -0.48)	0
	Mice and Rats	6	68	68	-2.53 (-3.79 to -1.27)	-2.04 (-2.50 to -1.58)	85
	Induced						
	Chemotherapy	5	48	48	-2.20 (-3.45 to -0.95)	-1.64 (-2.15 to -1.12)	81
	Autoimmune	1	20	20	-3.81 (-4.88 to -2.73)	-3.81 (-4.88 to -2.73)	
	Source of MSCs						
	Human	3	42	42	-2.23 (-3.84 to -0.62)	-2.07 (-2.64 to -1.49)	87
	Animal	3	26	26	-3.08 (-5.82 to -0.33)	-1.99 (-2.77 to -1.20)	89
	Types of MSCs						
	UCMSCs	3	42	42	-2.23 (-3.84 to -0.62)	-2.07 (-2.64 to -1.49)	87
	BMMSCs	2	16	16	-4.21 (-8.90 to 0.47)	-3.47 (-4.81 to -2.13)	91
	ADMSCs	1	10	10	-1.21 (-2.18 to -0.24)	-1.21 (-2.18 to -0.24)	
	Dose of transplantation						
	10^5^	3	27	27	-1.23 (-1.83 to -0.63)	-1.23 (-1.83 to -0.63)	0
	10^6^	3	41	41	-3.90 (-6.10 to -1.70)	-3.23 (-3.96 to -2.50)	87
	Route of transplantation						
	IV	4	43	43	-2.32 (-3.84 to -0.80)	-1.61 (-2.15 to -1.07)	85
	IO	2	25	25	-2.96 (-4.82 to -1.09)	-3.23 (-4.13 to -2.33)	73
**FSH/LH**		3	23	23	-0.08 (-0.66 to 0.49)	-0.08 (-0.66 to 0.49)	0
	Animals						
	Rats	1	6	6	-0.06 (-1.20 to 1.07)	-0.06 (-1.20 to 1.07)	
	Mice	1	12	12	-0.10 (-0.90 to 0.71)	-0.10 (-0.90 to 0.71)	
	Mice and Rats	2	18	18	-0.08 (-0.74 to 0.57)	-0.08 (-0.74 to 0.57)	0
	Rabbits	1	5	5	-0.08 (-1.32 to 1.16)	-0.08 (-1.32 to 1.16)	
	Induced						
	Chemotherapy	3	23	23	-0.08 (-0.66 to 0.49)	-0.08 (-0.66 to 0.49)	0
	Source of MSCs						
	Human	3	23	23	-0.08 (-0.66 to 0.49)	-0.08 (-0.66 to 0.49)	0
	Types of MSCs						
	UCMSCs	3	23	23	-0.08 (-0.66 to 0.49)	-0.08 (-0.66 to 0.49)	0
	Dose of transplantation						
	10^6^	2	18	18	-0.08 (-0.74 to 0.57)	-0.08 (-0.74 to 0.57)	0
	10^7^	1	5	5	-0.08 (-1.32 to 1.16)	-0.08 (-1.32 to 1.16)	
	Route of transplantation						
	IV	3	23	23	-0.08 (-0.66 to 0.49)	-0.08 (-0.66 to 0.49)	0
**INHB**		2	10	10	23.51 (14.11 to 32.91)	23.51 (14.11 to 32.91)	0
	Animals						
	Rats	1	5	5	23.53 (10.23 to 36.84)	23.53 (10.23 to 36.84)	
	Mice	1	5	5	23.49 (10.21 to 36.77)	23.49 (10.21 to 36.77)	
	Mice and Rats	2	10	10	23.51 (14.11 to 32.91)	23.51 (14.11 to 32.91)	0
	Induced						
	Chemotherapy	2	10	10	23.51 (14.11 to 32.91)	23.51 (14.11 to 32.91)	0
	Source of MSCs						
	Human	2	10	10	23.51 (14.11 to 32.91)	23.51 (14.11 to 32.91)	0
	Types of MSCs						
	UCMSCs	2	10	10	23.51 (14.11 to 32.91)	23.51 (14.11 to 32.91)	0
	Dose of transplantation						
	10^6^	1	5	5	23.53 (10.23 to 36.84)	23.53 (10.23 to 36.84)	
	10^7^	1	5	5	23.49 (10.21 to 36.77)	23.49 (10.21 to 36.77)	
	Route of transplantation						
	IV	2	10	10	23.51 (14.11 to 32.91)	23.51 (14.11 to 32.91)	0
**Atretic follicles**		9	92	92	-1.71 (-3.08 to -0.33)	-1.69 (-2.09 to -1.28)	90
	Animals						
	Rats	4	41	41	-2.41 (-3.86 to -0.96)	-1.76 (-2.33 to -1.20)	81
	Mice	4	46	46	-0.25 (-3.32 to 2.82)	-1.49 (-2.10 to -0.88)	95
	Mice and Rats	8	87	87	-1.57 (-3.06 to -0.09)	-1.64 (-2.05 to -1.22)	91
	Rabbits	1	5	5	-2.83 (-4.84 to -0.81)	-2.83 (-4.84 to -0.81)	
	Induced						
	Chemotherapy	9	92	92	-1.71 (-3.08 to -0.33)	-1.69 (-2.09 to -1.28)	90
	Source of MSCs						
	Human	8	82	82	-1.27 (-2.62 to 0.07)	-1.54 (-1.95 to -1.13)	89
	Animal	1	10	10	-6.07 (-8.35 to -3.80)	-6.07 (-8.35 to -3.80)	
	Types of MSCs						
	UCMSCs	7	67	67	-1.21 (-2.89 to 0.47)	-1.68 (-2.16 to -1.19)	91
	BMMSCs	1	10	10	-6.07 (-8.35 to -3.80)	-6.07 (-8.35 to -3.80)	
	PMSCs	1	15	15	-1.18 (-1.97 to -0.40)	-1.18 (-2.06 to -1.16)	
	Dose of transplantation						
	10^5^	1	9	9	8.00 (4.90 to 11.10)	8.00 (4.90 to 11.10)	
	10^6^	6	63	63	-2.70 (-4.12 to -1.28)	-2.06 (-2.56 to -1.57)	87
	10^7^	2	20	20	-1.73 (-3.25 to -0.21)	-1.40 (-2.13 to -0.67)	55
	Route of transplantation						
	IV	8	83	83	-2.45 (-3.51 to -1.38)	-1.86 (-2.26 to -1.45)	83
	IO	1	9	9	8.00 (4.90 to 11.10)	8.00 (4.90 to 11.10)	
**Antral follicles**		6	59	54	2.85 (0.58 to 5.11)	1.90 (1.33 to 2.48)	93
	Animals						
	Rats	3	36	31	2.76 (-0.63 to 6.15)	2.06 (1.32 to 2.80)	95
	Mice	3	23	23	3.13 (-0.99 to 7.26)	1.65 (0.72 to 2.58)	94
	Mice and Rats	6	59	54	2.85 (0.58 to 5.11)	1.90 (1.33 to 2.48)	93
	Induced						
	Chemotherapy	5	54	49	3.64 (1.24 to 6.04)	2.55 (1.91 to 3.19)	91
	Autoimmune	1	5	5	-0.90 (-2.24 to 0.44)	-0.90 (-2.24 to 0.44)	
	Source of MSCs						
	Human	4	38	35	4.03 (0.31 to 7.74)	2.50 (1.65 to 3.35)	94
	Animal	2	21	19	1.02 (-2.13 to 4.18)	1.38 (0.59 to 2.18)	93
	Types of MSCs						
	UCMSCs	4	38	35	4.03 (0.31 to 7.74)	2.50 (1.65 to 3.35)	94
	BMMSCs	1	5	5	-0.62 (-1.90 to 0.67)	-0.62 (-1.90 to 0.67)	
	ADMSCs	1	16	14	2.61 (1.60 to 3.61)	2.61 (1.60 to 3.61)	
	Dose of transplantation						
	10^5^	1	6	6	7.75 (3.80 to 11.70)	7.75 (3.80 to 11.70)	
	10^6^	5	53	48	2.17 (-0.14 to 4.48)	1.78 (1.19 to 2.36)	93
	Route of transplantation						
	IV	1	12	12	3.59 (2.22 to 4.96)	3.59 (2.22 to 4.96)	
	IO	5	47	42	2.74 (0.04 to 5.44)	1.54 (0.90 to 2.18)	93
**Growing follicles**		5	58	58	2.08 (1.19 to 2.98)	1.80 (1.33 to 2.26)	68
	Animals						
	Rats	3	38	38	2.55 (0.95 to 4.16)	1.96 (1.37 to 2.55)	82
	Mice	1	15	15	1.43 (0.61 to 2.24)	1.43 (0.61 to 2.24)	
	Mice and Rats	4	53	53	2.13 (1.08 to 3.18)	1.78 (1.30 to 2.25)	76
	Rabbits	1	5	5	2.05 (0.35 to 3.74)	2.05 (0.35 to 3.74)	
	Induced						
	Chemotherapy	5	58	58	2.08 (1.19 to 2.98)	1.80 (1.33 to 2.26)	68
	Source of MSCs						
	Human	4	48	48	1.61 (1.13 to 2.08)	1.61 (1.13 to 2.08)	0
	Animal	1	10	10	5.29 (3.26 to 7.32)	5.29 (3.26 to 7.32)	
	Types of MSCs						
	UCMSCs	4	48	48	1.61 (1.13 to 2.08)	1.61 (1.13 to 2.08)	0
	BMMSCs	1	10	10	5.29 (3.26 to 7.32)	5.29 (3.26 to 7.32)	
	Dose of transplantation						
	10^6^	4	53	53	2.13 (1.08 to 3.18)	1.78 (1.30 to 2.25)	76
	10^7^	1	5	5	2.05 (0.35 to 3.74)	2.05 (0.35 to 3.74)	
	Route of transplantation						
	IV	5	58	58	2.08 (1.19 to 2.98)	1.80 (1.33 to 2.26)	68
**Mature follicles**		5	56	53	3.10 (1.86 to 4.34)	2.62 (2.06 to 3.19)	76
	**Animals**						
	Rats	5	56	53	3.10 (1.86 to 4.34)	2.62 (2.06 to 3.19)	76
	Induced						
	Chemotherapy	3	33	33	2.69 (1.00 to 4.38)	2.18 (1.51 to 2.85)	81
	Autoimmune	2	23	20	3.76 (2.69 to 4.83)	3.76 (2.69 to 4.83)	0
	**Source of MSCs**						
	Human	4	46	43	2.64 (1.52 to 3.76)	2.40 (1.81 to 2.99)	69
	Animal	1	10	10	5.29 (3.26 to 7.32)	5.29 (3.26 to 7.32)	
	Types of MSCs						
	UCMSCs	3	31	28	2.97 (1.32 to 4.63)	2.72 (1.93 to 3.50)	75
	BMMSCs	1	10	10	5.29 (3.26 to 7.32)	5.29 (3.26 to 7.32)	
	PMSCs	1	15	15	1.98 (1.09 to 2.88)	1.98 (1.09 to 2.88)	
	Dose of transplantation						
	10^6^	4	41	38	3.50 (1.86 to 5.13)	3.05 (2.32 to 3.78)	78
	10^7^	1	15	15	1.98 (1.09 to 2.88)	1.98 (1.09 to 2.88)	
	Route of transplantation						
	IV	4	47	47	2.90 (1.53 to 4.27)	2.49 (1.90 to 3.08)	79
	IO	1	9	6	4.15 (2.14 to 6.16)	4.15 (2.14 to 6.16)	
**Corpus leteum**		5	44	41	1.02 (-1.47 to 3.51)	0.27 (-0.32 to 0.86)	93
	Animals						
	Rats	4	39	36	1.96 (-0.81 to 4.72)	0.56 (-0.06 to 1.17)	94
	Rabbits	1	5	5	-2.85 (-4.88 to -0.82)	-2.85 (-4.88 to -0.82)	
	Induced						
	Chemotherapy	3	21	21	-1.18 (-2.15 to -0.21)	-1.05 (-1.73 to -0.36)	42
	Autoimmune	2	23	20	5.19 (0.99 to 9.39)	3.81 (2.68 to 4.93)	82
	Source of MSCs						
	Human	5	44	41	1.02 (-1.47 to 3.51)	0.27 (-0.32 to 0.86)	93
	Types of MSCs						
	UCMSCs	5	44	41	1.02 (-1.47 to 3.51)	0.27 (-0.32 to 0.86)	93
	Dose of transplantation						
	10^6^	4	39	36	1.96 (-0.81 to 4.72)	0.56 (-0.06 to 1.17)	94
	10^7^	1	5	5	-2.85 (-4.88 to -0.82)	-2.85 (-4.88 to -0.82)	
	Route of transplantation						
	IV	4	35	35	-0.22 (-2.56 to 2.11)	0.04 (-0.56 to 0.63)	93
	IO	1	9	6	7.65 (4.30 to 11.00)	7.65 (4.30 to 11.00)	
**Follicles**		3	30	30	3.05 (-0.58 to 6.69)	2.51 (1.66 to 3.37)	94
	Animals						
	Rats	3	30	30	3.05 (-0.58 to 6.69)	2.51 (1.66 to 3.37)	94
	Induced						
	Chemotherapy	2	10	10	1.06 (0.08 to 2.05)	1.06 (0.08 to 2.05)	0
	Autoimmune	1	20	20	7.03 (5.30 to 8.77)	7.03 (5.30 to 8.77)	
	Source of MSCs						
	Human	1	20	20	7.03 (5.30 to 8.77)	7.03 (5.30 to 8.77)	
	Animal	2	10	10	1.06 (0.08 to 2.05)	1.06 (0.08 to 2.05)	0
	Types of MSCs						
	UCMSCs	1	20	20	7.03 (5.30 to 8.77)	7.03 (5.30 to 8.77)	
	BMMSCs	2	10	10	1.06 (0.08 to 2.05)	1.06 (0.08 to 2.05)	0
	Dose of transplantation						
	10^6^	2	25	25	3.85 (-2.33 to 10.03)	3.00 (1.95 to 4.04)	97
	10^7^	1	5	5	1.51 (0.01 to 3.01)	1.51 (0.01 to 3.01)	
	Route of transplantation						
	IO	3	30	30	3.05 (-0.58 to 6.69)	2.51 (1.66 to 3.37)	94
**Early antral**		2	15	15	1.00 (0.23 to 1.78)	1.00 (0.23 to 1.78)	0
	Animals						
	Rats	2	15	15	1.00 (0.23 to 1.78)	1.00 (0.23 to 1.78)	0
	Induced						
	Chemotherapy	2	15	15	1.00 (0.23 to 1.78)	1.00 (0.23 to 1.78)	0
	Source of MSCs						
	Human	2	15	15	1.00 (0.23 to 1.78)	1.00 (0.23 to 1.78)	0
	Types of MSCs						
	UCMSCs	2	15	15	1.00 (0.23 to 1.78)	1.00 (0.23 to 1.78)	0
	Dose of transplantation						
	10^5^	1	3	3	0.65 (-1.07 to 2.37)	0.65 (-1.07 to 2.37)	
	10^6^	1	12	12	1.09 (0.22 to 1.96)	1.09 (0.22 to 1.96)	
	Route of transplantation						
	IV	1	12	12	1.09 (0.22 to 1.96)	1.09 (0.22 to 1.96)	
	IO	1	3	3	0.65 (-1.07 to 2.37)	0.65 (-1.07 to 2.37)	
**Estruc cycle**		7	90	90	0.00 (-1.65 to 1.66)	0.54 (0.17 to 0.91)	94
	Animals						
	Rats	4	70	70	0.59 (-1.57 to 2.75)	0.88 (0.46 to 1.29)	96
	Mice	3	20	20	-0.85 (-3.63 to 1.93)	-0.78 (-1.60 to 0.05)	90
	Mice and Rats	7	90	90	0.00 (-1.65 to 1.66)	0.54 (0.17 to 0.91)	94
	Induced						
	Chemotherapy	5	40	40	-0.62 (-2.68 to 1.44)	-0.17 (-0.75 to 0.40)	92
	Autoimmune	1	20	20	-0.31 (-0.93 to 0.31)	-0.31 (-0.93 to 0.31)	
	Radiation	1	30	30	3.18 (2.40 to 3.96)	3.18 (2.40 to 3.96)	
	Source of MSCs						
	Human	6	60	60	-0.54 (-1.98 to 0.90)	-0.24 (-0.66 to 0.19)	90
	Animal	1	30	30	3.18 (2.40 to 3.96)	3.18 (2.40 to 3.96)	
	Types of MSCs						
	UCMSCs	4	46	46	-0.66 (-2.32 to 1.00)	-0.20 (-0.67 to 0.26)	90
	BMMSCs	1	30	30	3.18 (2.40 to 3.96)	3.18 (2.40 to 3.96)	
	AFMSCs	1	8	8	-2.46 (-3.84 to -1.07)	-2.46 (-3.84 to -1.07)	
	CPMSCs	1	6	6	2.01 (0.51 to 3.51)	2.01 (0.51 to 3.51)	
	Dose of transplantation						
	10^5^	1	6	6	-3.07 (-4.95 to -1.19)	-3.07 (-4.95 to -1.19)	
	10^6^	6	84	84	0.46 (-1.24 to 2.16)	0.69 (0.31 to 1.07)	95
	Route of transplantation						
	IV	5	64	64	0.61 (-1.54 to 2.76)	1.27 (0.79 to 1.75)	95
	IO	2	26	26	-1.54 (-4.23 to 1.15)	-0.58 (-1.18 to -0.01)	87

UC-MSCs, umbilical cord mesenchymal stem cells; BMMSCs, bone marrow mesenchymal stem cells; MenSCs, Menstrual blood mesenchymal stem cells; PMSCs, placenta mesenchymal stem cells; CPMSCs, chorionic plate mesenchymal stem cells; ADMSCs, adipose mesenchymal stem cell; AFMSCs, amniotic fluid mesenchymal stem cells; IV, intravenously injected; IO, intra ovary injected; LH, luteinizing hormone; FSH, follicle-stimulating hormone; E_2_, estradiol; AMH, anti Mullerian hormone; INHB, inhibin B; SMD, standardized mean difference.

## Discussion

This systematic review and meta-analysis examining 37 studies (1,079 animals) found that mesenchymal stem cell transplantation might effectively improve ovarian function in premature ovarian failure in animal model studies.

According to the source of the isolated tissue, MSCs can be classified into different types. Multiple previous studies indicated that MSC transplantation was effective in repairing tissue damage, including neurodegenerative diseases, skin injury, cardiovascular disease, bone damage, and diabetes (46–49). For POF, MSC therapy had promising potential for clinical use but some safety and manufacturing issues have kept it mostly in the animal model stage.

In preclinical studies, MSCs appeared to be able to improve certain functional measures of damaged ovaries, including sex hormones and the number of follicles at different stages, in POF animal models. However, MSCs from different tissues produced different effects. One study has shown (11) that primary and secondary follicles increased, atretic follicles decreased, and ovarian volume also can increase after UC-MSC treatment in POF mice. Another study found that BM-MSCs could regulate and activate ovarian function and regain fertility in a radiation-induced POF rat model (10). Meanwhile, serum hormone levels, including E2 and FSH, were restored after the transplantation of CP-MSC in a chemotherapy-induced POF mouse model (13). Overall, our results are generally consistent with previous research and showed that different MSCs from different sources are different in repairing ovarian function in this study. In addition to the properties of specialized cells, the sample size and included studies are also important and affect treatment outcomes, and small samples often produced false negative results.

It is important to note that, from the analysis of the 37 studies, it is suggested that MSC transplantation enhanced the primordial follicle formation and follicle growth up to the secondary follicle stage, but not to the antral follicle stage, thus contributing to hormonal status as far as estrogen levels are concerned while not contributing to re-establishing the estrus cycle. The reason for this may be the limitations of the original articles concerning their methodological quality or their small sample size. Meanwhile, the significant changes found in the analyses regarding different parameters should be evaluated and commented on with respect to the duration of the effect and observation period.

Although they have been shown to be safe in animal models, the risk cannot be ignored concerning MSCs being transplanted into patients. Several small-sample clinical trials indicated that ovarian function and fertility could improve in women with POF after MSC therapy. In their phase I study (1), the authors found MSCs were safe to use for more than a year, and menstruation also can resume for POF patients. Other clinical results (3, 50, 51) also showed that transplantation of MSCs could improve ovarian features, including increasing the number of follicles and improving ovarian hormone production, in POF patients. In these human studies, it seems that the improvement is somewhat transient or temporary. Thus, it is important to have a similar conclusion in animal studies and provide some evidence and guidance to the readers for the next step.

In this comprehensive, thorough, and complete systematic review and meta-analysis study, there is further evidence, obtained by increasing the sample size in POF animal models, that MSC treatment could rescue ovarian function. The results of this study will provide more powerful preclinical medical evidence support for the application of MSCs in high-quality clinical trials. Our study further demonstrates the ability of MSCs to treat POF. However, it is still a tough task for the transformation from animal to human. More detailed mechanisms of MSC treatment for POF need to be discovered in the future. In addition, continuous follow-ups are needed to evaluate the long-term effects of this new intervention.

There are still several limitations of this study that need consideration. First, we only searched the commonly used English and Chinese databases, which would lead to some language bias. Second, the included studies were generally of low methodological quality, and the sample size of each study was small. Third, some outcomes could not be subgroup analyzed because of small sample sizes. In addition, the various sources of MSCs and animal species could limit the value of MSCs in the treatment of premature ovarian failure.

## Conclusions

In summary, this systematic review and meta-analysis indicated that MSCs might exert therapeutic effects on animal models of POF, and these effects might be associated with improving the disorder of the sexual cycle, modulating the serum hormone expression to a better state, and restoring ovarian function.

## Data availability statement

The original contributions presented in the study are included in the article/**Supplementary Material**. Further inquiries can be directed to the corresponding author.

## Author contributions

DF participated in the design and coordination of the study. CG conceived the study and drafted the manuscript. YM, YS, LL, GL, HL, WM, LS, and WW searched for the studies and collected and analyzed the data. WW, QW, and LW participated in the design of this study and edited the manuscript. DF, LL, and YM did the data management and analyzed the data. All authors contributed to the article and approved the submitted version.

## Supplementary material

Supplementary Table 1 PRISMA Checklist.

Supplementary Table 2 Study quality and risk of bias.

Supplementary Data Sheet 1 Study protocol.

Supplementary Data Sheet 2 Search strategy.

Supplementary Data Sheet 3
**Supplementary Figures 1 to 5**.
