# Supplementary material for: Mesenchymal stem cells therapy improves ovarian function in premature ovarian failure: a systematic review and meta-analysis based on preclinical studies
Source: Front Endocrinol (Lausanne). 2023 Jul 6;14:1165574. doi: 10.3389/fendo.2023.1165574 (PMC10361781; doi:10.3389/fendo.2023.1165574)
Supplement: Supplementary Table 2 — Study quality and risk of bias. [file Table_2.docx]

Supplementary Table 2. The results of included study quality and risk of bias

| Reference | Selection bias | | | Performance bias | | Detection bias | | Attrition bias | Reporting bias | Other |
| --- | --- | --- | --- | --- | --- | --- | --- | --- | --- | --- |
|  | Sequence generation | Baseline characteristics | Allocation concealment | Random housing | Blinding | Random outcome assessment | Blinding | Incomplete outcome data | Selective outcome reporting | Other sources of bias |
| Yan PY, 2022 | U | Y | N | U | U | U | U | N | N | N |
| Zhao SY, 2022 | U | Y | N | U | U | U | U | N | N | N |
| Wu Y, 2021 | U | Y | N | U | U | U | U | N | N | N |
| Deng TR, 2021 | U | Y | N | U | U | U | U | N | N | N |
| El-Derany MO, 2021 | U | Y | N | U | U | U | U | N | N | N |
| Zhang L, 2021 | Y | Y | N | U | U | U | U | N | N | N |
| Zhang XY, 2021 | U | Y | N | U | U | U | U | N | N | N |
| Li Q, 2020 | U | Y | N | U | U | U | U | N | N | N |
| Wang Z, 2020 | U | Y | N | U | U | U | U | N | N | N |
| Xu YY, 2020 | U | Y | N | U | U | U | U | N | N | N |
| Zhang J, 2020 | Y | Y | N | U | U | U | U | N | N | N |
| Hou QN, 2019 | U | Y | N | U | U | U | U | N | N | N |
| Lin HW, 2019 | U | Y | N | U | U | U | U | N | N | N |
| Manshadi MD, 2019 | U | Y | N | U | U | U | U | N | N | N |
| Tan L, 2019 | U | Y | N | U | U | U | U | N | N | N |
| Yang YJ, 2019 | U | Y | N | U | U | U | U | N | N | N |
| Zheng Q, 2019 | U | Y | N | U | U | U | U | N | N | N |
| Zhao Y, 2019 | Y | Y | N | U | U | U | U | N | N | N |
| Zhuang YQ, 2019 | Y | Y | N | U | U | U | U | N | N | N |
| Li J, 2018 | U | Y | N | U | U | U | U | N | N | N |
| Li XR, 2018 | U | Y | N | U | U | U | U | N | N | N |
| Wang LL, 2018 | U | Y | N | U | U | U | U | N | N | N |
| Yin N, 2018 | U | Y | N | U | U | U | U | N | N | N |
| Zhang LL, 2018 | Y | Y | N | U | U | U | U | N | N | N |
| Badawy A, 2017 | U | Y | N | U | U | U | U | N | N | N |
| Jia XC, 2017 | U | Y | N | U | U | U | U | N | N | N |
| Wu Q, 2017 | Y | Y | N | U | U | U | U | N | N | N |
| Elfayomy AK, 2016 | U | Y | N | U | U | U | U | N | N | N |
| Gabr H, 2016 | U | Y | N | U | U | U | U | N | N | N |
| Song D, 2016 | U | Y | N | U | U | U | U | N | N | N |
| Su J, 2016 | U | Y | N | U | U | U | U | N | N | N |
| Qin JJ, 2015 | U | Y | N | U | U | U | U | N | N | N |
| Ye XF, 2015 | U | Y | N | U | U | U | U | N | N | N |
| Fu XF, 2013 | U | Y | N | U | U | U | U | N | N | N |
| Li J, 2012 | U | Y | N | U | U | U | U | N | N | N |
| Wang Y, 2011 | U | Y | N | U | U | U | U | N | N | N |
| Fu X, 2008 | U | Y | N | U | U | U | U | N | N | N |

N: No, U: Unclear, Y: Yes.
