# Supplementary material for: Mesenchymal stem cells therapy improves ovarian function in premature ovarian failure: a systematic review and meta-analysis based on preclinical studies
Source: Front Endocrinol (Lausanne). 2023 Jul 6;14:1165574. doi: 10.3389/fendo.2023.1165574 (PMC10361781; doi:10.3389/fendo.2023.1165574)
Supplement: Supplementary Data Sheet 1 — Study protocol. [file DataSheet_1.docx]

**Supplementary Data Sheet 1**:

The study protocol of Mesenchymal Stem Cell Therapy Improves Ovarian Function for Premature Ovarian Failure: A Systematic review and meta-analysis based on preclinical studies

**Review title:** Mesenchymal Stem Cell Therapy Improve Ovarian Function for Premature Ovarian Failure: A Systematic review and meta-analysis based on preclinical studies

**Review question:** To explore the potential of using mesenchymal stem cell (MSC) therapy in premature ovarian failure (POF) in animal models.

**Condition being studied:** The efficacy of MSC therapy for POF has been investigated in animal experiments but individual studies with a small sample size cannot be used to draw a clear conclusion.

**Searches:** A systematic literature search will be performed in Pubmed, Embase, Web of Science, VIP information database, Wanfang Data Information Site, Chinese Biomedical Literature (CBM), and China National Knowledge Infrastructure (CNKI) from the inception of each database until August 2022. The initial search was performed in PubMed, including the following search terms: “stem cell”; “MSC”; “mesenchymal stem cells”, “premature ovarian failure”; “POF”; “POI”; and “premature ovarian insufficiency”. The corresponding search terms in the Chinese language were used to search the Chinese databases. No restrictions for language or geographic location were applied.

**Participants:** POF model animals. The animal participants consisted of rats, mice, rabbits, and other animals.

**Exposures:** The exposure was MSC transplantation, including both human and animal MSCs, from umbilical cord MSCs (UC-MSCs), bone marrow MSCs (BMMSCs), menstrual blood MSCs (MenSCs), placenta MSCs (PMSCs), chorionic plate MSCs (CPMSCs), adipose MSCs (ADMSCs), amniotic fluid MSCs (AFMSCs), and other tissues-derived MSCs.

**Controls:** The control was placebo transplantation, such as 0.9% saline and phosphate-buffered saline.

**Types of study to be included:** The type of study was a randomized controlled study. Study types to be excluded were reviews, case studies, observational studies, and human studies. If they were duplicates, studies were excluded.

**Main outcomes:** The primary outcomes were estradiol (E2), follicle-stimulating hormone (FSH), primary follicles, and secondary follicles.

**Additional outcomes:** The secondary outcomes were other sex hormone outcomes, such as anti-Mullerian hormone (AMH), luteinizing hormone (LH), FSH/LH, inhibin B (INHB), and other follicle outcomes, such as primordial follicles, growing follicle, antral follicles, mature follicles, atretic follicles, corpus leteum, follicles, early antral and estruc cycle.

**Measures of effect:** The principal summary measures reported by studies were standardized mean difference (SMD) and 95% confidence interval (95% CI), and p-values.

**Data extraction:** Data from the included studies will be extracted into structured data tables. Extracted data included: study author, year and country of publication, sample size, type and dose of MSCs, type of animals, and assessment of outcomes. If multiple measurements were reported within a period, the closest to week 4 will be extracted. Data extraction will be completed by 2 independent authors. In case of missing data in a relevant article, the corresponding and/or primary authors will be contacted for additional information.

**Risk of bias assessment:** The methodologic quality in each included study will be evaluated by the SYRCLE risk of bias tool for animal studies. Two reviewers will independently assess the quality and mediated by the third reviewer for each included study.

**Strategy for data synthesis:** Data analysis and forest plots will be completed using Review Manager 5.4.1 (Copenhagen: The Nordic Cochrane Center, Cochrane Collaboration, 2020) and Stata version 12 (StataCorp, College Station, TX, USA). We will calculate the standardized mean difference (SMD) and 95% confidence interval (95% CI) for each separate intervention-control comparison group. Random or fixed effects models will be used for the meta-analyses based on heterogeneity. Heterogeneity between studies will be assessed by the *I^2^* statistics. We will define low, moderate, and high heterogeneity according to *I^2^* cut-offs of 25%, 50%, and 75%, respectively. If there are more than 10 studies for one outcome, potential publication biases will be also assessed graphically by using a funnel plot. Egger’s regression tests for the likelihood of publication bias will be performed.

**Analysis of subgroups or subsets:** We will perform subgroup analyses for animal species (rats, mice, rabbits), induced (chemotherapy, autoimmune, radiation), source of MSCs (human, animal), and types of MSCs (UCMSCs, BMMSCs, AFMSCs, CPMSCs, MenSCs, ADMSCs, PMSCs).
