# Supplementary material for: Mesenchymal stem cells therapy improves ovarian function in premature ovarian failure: a systematic review and meta-analysis based on preclinical studies
Source: Front Endocrinol (Lausanne). 2023 Jul 6;14:1165574. doi: 10.3389/fendo.2023.1165574 (PMC10361781; doi:10.3389/fendo.2023.1165574)
Supplement: Supplementary Data Sheet 2 — Search strategy. [file DataSheet_2.docx]

Search strategy

(‘‘menopause, premature’’[MeSH] OR (‘‘menopause’’[All Fields] AND ‘‘premature’’[All Fields]) OR ‘‘premature menopause’’[All Fields] OR (‘‘premature’’[All Fields] AND ‘‘ovarian’’[All Fields] AND ‘‘failure’’[All Fields]) OR ‘‘premature ovarian failure’’[All Fields]) AND ("cell- and tissue-based therapy"[MeSH] OR "stem cells"[MeSH] OR "cell therapy"[All Fields] OR "cellular therapy"[All Fields] OR "cell transplantation"[All Fields] OR "cellular transplantation"[All Fields] OR "cell delivery"[All Fields] OR "cellular delivery"[All Fields] OR "cell infusion"[ All Fields] OR "cellular infusion"[All Fields] OR "stem cell"[All Fields] OR "stem cells"[All Fields])
