# Supplementary material for: Mesenchymal stem cells therapy improves ovarian function in premature ovarian failure: a systematic review and meta-analysis based on preclinical studies
Source: Front Endocrinol (Lausanne). 2023 Jul 6;14:1165574. doi: 10.3389/fendo.2023.1165574 (PMC10361781; doi:10.3389/fendo.2023.1165574)
Supplement: Supplementary Data Sheet 3 — Supplementary Figures 1 to 5 . [file DataSheet_3.docx]

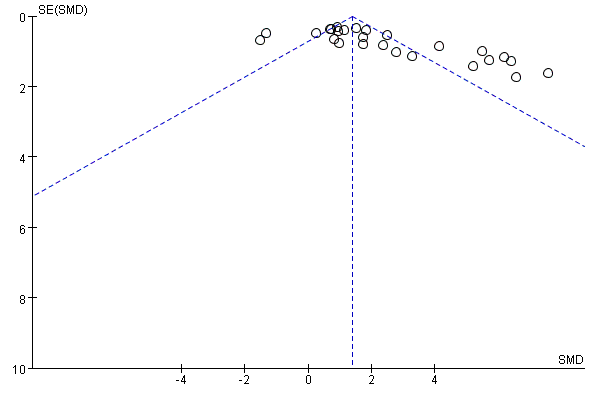


Supplementary Figure 1. Funnel plot of E2.


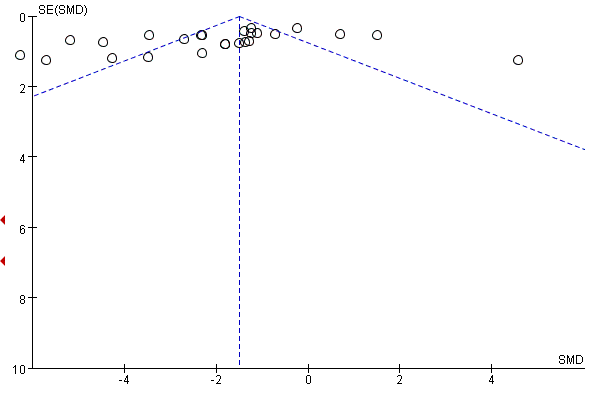


Supplementary Figure 3. Funnel plot of FSH.


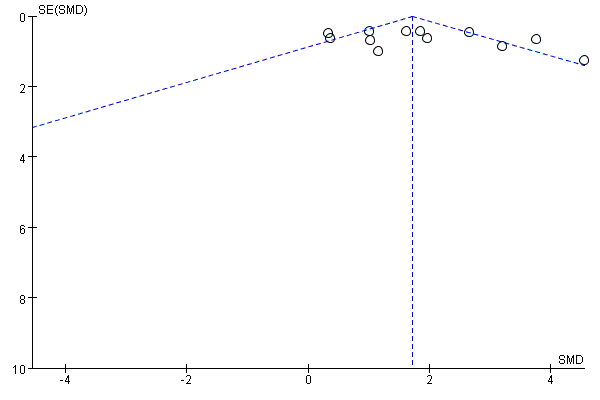
Supplementary Figure 2. Funnel plot of Primary follicles.


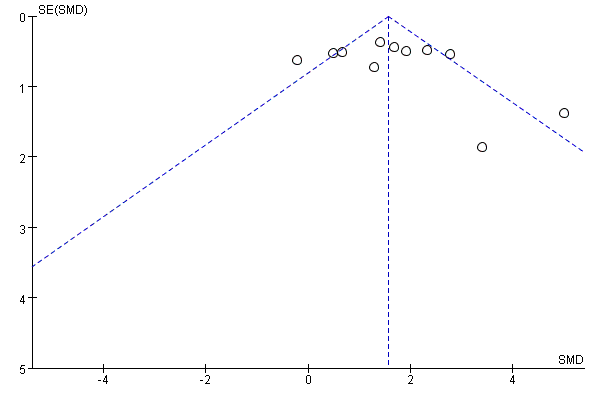


Supplementary Figure 4. Funnel plot of Secondary follicles.


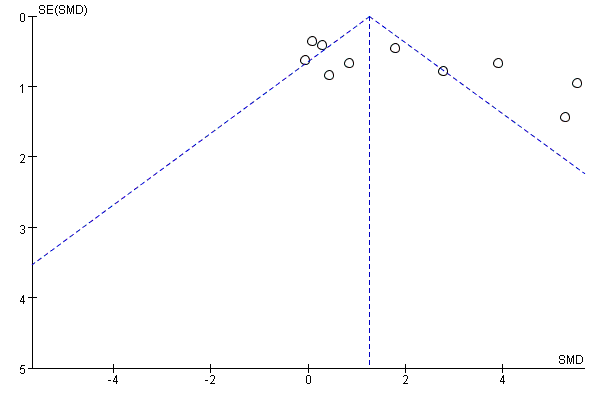
Supplementary Figure 5. Funnel plot of Primordial follicles.
